# Study of a group of infants with risks of hearing impairment and mental difficulties

**DOI:** 10.1192/j.eurpsy.2025.764

**Published:** 2025-08-26

**Authors:** Y. Sidneva, O. Lipatova, A. Zakrepina

**Affiliations:** 1 rehabilitation, Clinical and Research Institute of Emergency Pediatric Surgery and Traumatology (CIEPST); 2 neuropsychiatry, National Medical Research Center of Neurosurgery named after N.N. Burdenko; 3 defectology, Federal State Budgetary Scientific Institution Institute of Correctional Pedagogics /FGBNU ICP/, Moscow, Russian Federation

## Abstract

**Introduction:**

One of the little-studied nosologies with severe delay in psychomotor development in the first year of life is children with risks of damage to the auditory analyzer, which requires the development of an adapted method for diagnosing impaired auditory function and taking into account some external factors that do not depend on the state of the child’s auditory function.

**Objectives:**

to study the characteristics of the auditory function in infants at risk for hearing impairment with delayed psychomotor development.

**Methods:**

60 children (4-11 months) with suspected hearing loss were examined (Federal State Budgetary Scientific Institution “Institute of Correctional Pedagogics”); interview with parents; psychiatric and pedagogical examination.

**Results:**

Three groups of children were identified depending on the level of psychomotor development (a level close to the norm, with a slight delay of 1-3 months, with a significant delay - from 4 months):

*The first group (34%)* included children admitted with suspected hearing loss, with a level of psychomotor development close to the norm, without concomitant developmental disorders. All children in this group were found to have significant and minor hearing loss. In two children, the hearing condition (according to the better hearing ear) was assessed as normal with atresia of the auditory canal on one side.

*The second group (50%)* consisted of children with suspected hearing loss, whose psychomotor development was delayed by 1-3 months. Most of them had significant hearing loss. In two children, the suspicion of hearing loss was not confirmed.

*The third group (16%)* included children with suspected hearing loss with a significant delay in psychomotor development for more than 4 months. Half of them (8%) had disharmonious development. Most had significant hearing loss.

**Conclusions:**

given that each group included children with significant hearing loss and deafness, it became obvious that their psychomotor development level is independent of their hearing status.

The identified risks of sensory impairments combined with a pronounced delay in psychomotor development in the first year of life necessitate a search for markers of these disorders, and above all, factors and conditions that affect their manifestation and the dynamics of psychomotor development in children in the first year of life; presumably, this may be the child’s social environment.

**Disclosure of Interest:**

None Declared

